# Sex Determining Region Y-Box 2 (SOX2) Is a Potential Cell-Lineage Gene Highly Expressed in the Pathogenesis of Squamous Cell Carcinomas of the Lung

**DOI:** 10.1371/journal.pone.0009112

**Published:** 2010-02-09

**Authors:** Ping Yuan, Humam Kadara, Carmen Behrens, Ximing Tang, Denise Woods, Luisa M. Solis, Jiaoti Huang, Monica Spinola, Wenli Dong, Guosheng Yin, Junya Fujimoto, Edward Kim, Yang Xie, Luc Girard, Cesar Moran, Waun Ki Hong, John D. Minna, Ignacio I. Wistuba

**Affiliations:** 1 Department of Pathology, The University of Texas MD Anderson Cancer Center, Houston, Texas, United States of America; 2 Department of Thoracic/Head and Neck Medical Oncology, The University of Texas MD Anderson Cancer Center, Houston, Texas, United States of America; 3 Department of Biostatistics, The University of Texas MD Anderson Cancer Center, Houston, Texas, United States of America; 4 Department of Pathology and Laboratory Medicine, David Geffen School of Medicine, The University of California Los Angeles, Los Angeles, California, United States of America; 5 The Hamon Center for Therapeutic Oncology, The University of Texas Southwestern Medical Center, Dallas, Texas, United States of America; 6 Department of Biostatistics, The University of Texas Southwestern Medical Center, Dallas, Texas, United States of America; 7 Department of Internal Medicine, The University of Texas Southwestern Medical Center, Dallas, Texas, United States of America; 8 Department of Pharmacology, The University of Texas Southwestern Medical Center, Dallas, Texas, United States of America; University of Minnesota, United States of America

## Abstract

**Background:**

Non-small cell lung cancer (NSCLC) represents the majority (85%) of lung cancers and is comprised mainly of adenocarcinomas and squamous cell carcinomas (SCCs). The sequential pathogenesis of lung adenocarcinomas and SCCs occurs through dissimilar phases as the former tumors typically arise in the lung periphery whereas the latter normally arise near the central airway.

**Methodology/Principal Findings:**

We assessed the expression of SOX2, an embryonic stem cell transcriptional factor that also plays important roles in the proliferation of basal tracheal cells and whose expression is restricted to the main and central airways and bronchioles of the developing and adult mouse lung, in NSCLC by various methodologies. Here, we found that *SOX2* mRNA levels, from various published datasets, were significantly elevated in lung SCCs compared to adenocarcinomas (all p<0.001). Moreover, a previously characterized *OCT4/SOX2/NANOG* signature effectively separated lung SCCs from adenocarcinomas in two independent publicly available datasets which correlated with increased *SOX2* mRNA in SCCs. Immunohistochemical analysis of various histological lung tissue specimens demonstrated marked nuclear SOX2 protein expression in all normal bronchial epithelia, alveolar bronchiolization structures and premalignant lesions in SCC development (hyperplasia, dysplasia and carcinoma *in situ*) and absence of expression in all normal alveoli and atypical adenomatous hyperplasias. Moreover, SOX2 protein expression was greatly higher in lung SCCs compared to adenocarcinomas following analyses in two independent large TMA sets (TMA set I, n = 287; TMA set II, n = 511 both p<0.001). Furthermore, amplification of *SOX2* DNA was detected in 20% of lung SCCs tested (n = 40) and in none of the adenocarcinomas (n = 17).

**Conclusions/Significance:**

Our findings highlight a cell-lineage gene expression pattern for the stem cell transcriptional factor SOX2 in the pathogenesis of lung SCCs and suggest a differential activation of stem cell-related pathways between squamous cell carcinomas and adenocarcinomas of the lung.

## Introduction

Lung cancer continues to be the leading cause of cancer-related deaths in the United States and worldwide with over one million deaths each year [1]. The majority of lung cancers (85%) are NSCLCs that include SCCs and adenocarcinomas [2]. The pathogenesis of NSCLC involves the accumulation of genetic and epigenetic alterations in a long multi-step process due in part to chronic exposure to carcinogens such as tobacco smoke. Few early changes that occur during NSCLC pathogenesis have been identified. For example, mutations in *KRAS* [3] and in the epidermal growth factor receptor (*EGFR*) [4] typically occur early in the development of lung adenocarcinomas, whereas amplification of *EGFR* and *PI3KCA* [2] and epigenetic inactivation of the p16 tumor suppressor [5] are more frequent in SCC pathogenesis relative to adenocarcinomas. Moreover, the developmental transcription factor Thyroid transcriptional factor 1 (*TITF1*) has been shown to be a lineage-survival oncogene over-expressed and amplified in lung adenocarcinoma development [6]. Despite recent progress in the delineation of cellular pathways aberrantly modulated in NSCLC, our understanding of the molecular changes occurring early in NSCLC pathogenesis is still lacking.

Given that the embryonic stem cell *SOX2* transcriptional factor [7] plays important roles in tracheal epithelial cells [8] and is only expressed in the main airways and non-branching bronchioles in the developing and adult mouse lung [9], we hypothesized that it may be a cell-lineage gene highly and specifically expressed in SCCs that originate from central and upper airway and bronchial epithelial cells relative to adenocarcinomas that typically arise from the lung periphery [10]. In this study, we found that *SOX2* mRNA was largely higher in lung SCCs relative to adenocarcinomas from various microarray datasets. Moreover, when we analyzed an *OCT4/SOX2/NANOG* stem cell gene signature previously characterized by Boyer *et al.* [11] in independent publicly available NSCLC datasets, the signature was found to effectively discriminate both major NSCLC subtypes. In addition, we found marked SOX2 protein expression only in the pathogenesis of SCC which was greatly higher in SCCs relative to lung adenocarcinomas following analyses of two independent and large tissue microarray (TMA) sets. Lastly, amplification of *SOX2* DNA assessed by quantitative PCR (qPCR) was evident in 20% of lung SCCs studied (n = 40) and was absent in all adenocarcinoma cases tested (n = 17).

## Results

### Elevated Expression of *SOX2* mRNA in Lung SCCs Relative to Adenocarcinomas in Various Microarray Datasets

**Figure 1:**
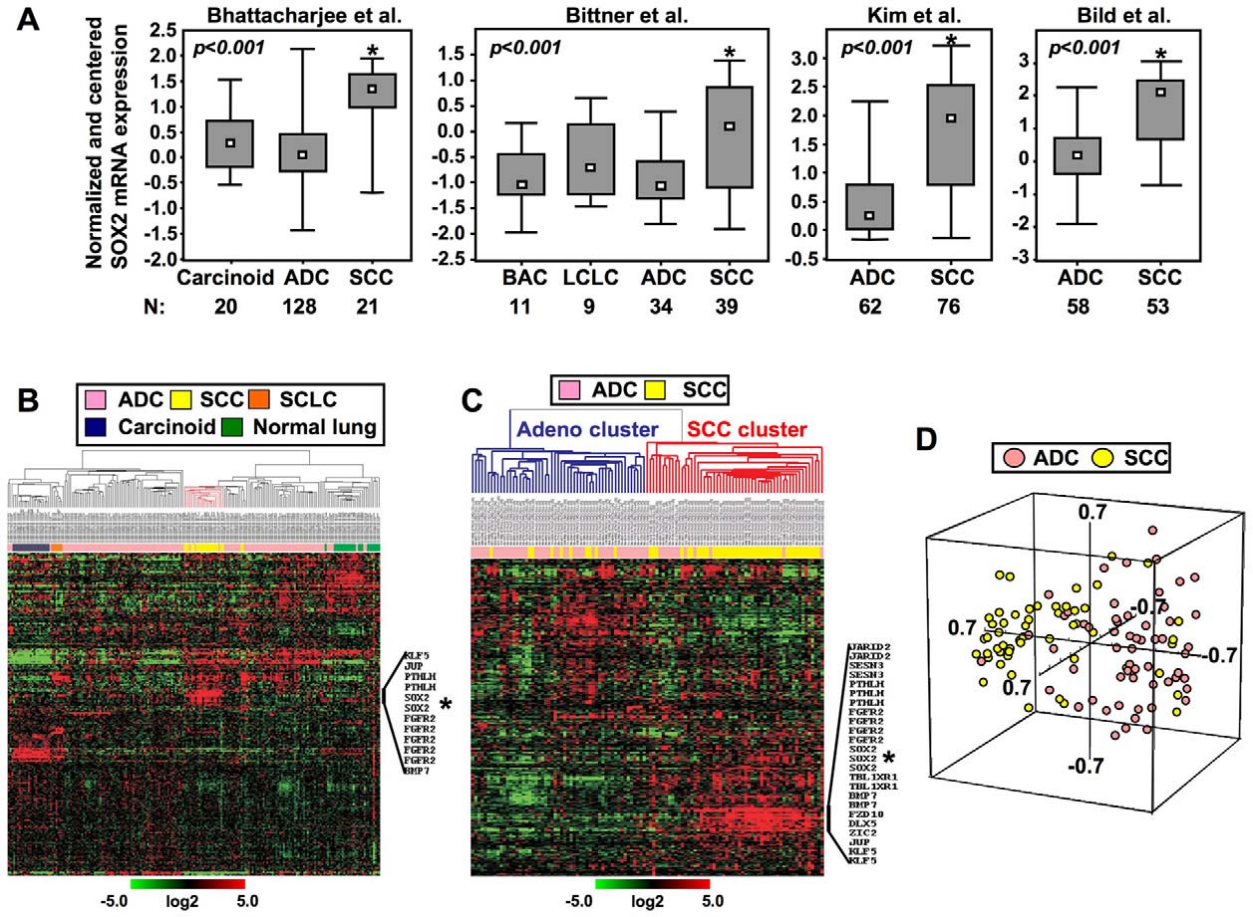
Increased expression of *SOX2* mRNA in lung SCCs relative to adenocarcinomas. **A.** Normalized centered *SOX2* mRNA expression levels downloaded from four published microarray data sets as described in the Methods. The lead author of each published microarray cohort data is indicated in each panel. The number of analyzed samples is indicated below each column bar. p-values represent statistical significance assessed by independent two-sided t-tests. **B.** Hierarchical cluster analysis with average linkage of the expression of the previously characterized *OCT4/SOX2/NANOG* signature [11] using present probe sets features in the array platforms of the datasets by Bhattacharjee *et al.* [12] (**B**), and Bild *et al.* [13] (**C**). Data are represented in a matrix format in which individual rows represent single gene features and columns represent experiments. High or low gene expression levels are indicated by red or green color, respectively as indicated by the log2 transformed scale bars. **D.** Principal component analysis of the signature in the Bild *et al.* dataset using metric centered correlation.

Analysis of *SOX2* mRNA expression in NSCLC samples of various publicly available datasets revealed the significant elevated expression of this stem cell-related transcriptional factor in lung SCCs compared to adenocarcinomas (all p<0.001) (Figure 1A). This differential expression pattern was also evident when microarray analysis was performed on FFPE tissue sections of NSCLC (p<0.001) (Figure S1).

### A Previously Characterized *OCT4/SOX2/NANOG* Signature Effectively Discriminates Lung SCCs from Adenocarcinomas

Since *SOX2* mRNA levels were largely higher in SCCs than adenocarcinomas of the lung, we questioned whether a *SOX2*-related signature can discriminate between the two subtypes of NSCLC. Using only gene features represented in the published dataset platforms, the previously characterized *OCT4/SOX2/NANOG* signature by Boyer *et al.* [11] was analyzed alone using human gene expression data from the studies by Bhattacharjee *et al* and Bild *et al* [12], [13]. In both datasets, the *OCT4/SOX2/NANOG* signature effectively separated lung tumors based on histology where most SCCs clustered alone (Figures 1B and 1C). In hierarchical cluster analysis of the Bild dataset, 43 out of 53 SCCs clustered together (Figure 1C) (p = 4.3×10^−10^ of the χ-test). The sensitivity and specificity for the classification of SCCs and lung adenocarcinomas were 0.81 and 0.78, respectively (Table S1). Moreover, the separation of SCCs from lung adenocarcinomas by the signature was also evident by principal component analysis in three-dimensional space (Figure 1D). In addition, *SOX2* expression in the cluster analyses was higher in lung SCCs relative to adenocarcinomas. Moreover, the expression of *SOX2* mRNA correlated with that of genes such as fibroblast growth factor receptor 2 (*FGFR2*) and parathyroid hormone-like hormone (*pTHLH*) in cluster analyses of both independent datasets (Figures 1B and 1C). In an attempt to validate these findings, we correlated the protein expression of SOX2 with that of FGFR2 by TMA immunohistochemistry analysis and found that nuclear SOX2 protein levels correlated significantly with cytoplasmic FGFR2 levels in lung SCC histological tissue specimens (Figure S2) but not in adenocarcinomas (data not shown).

### Differential Expression of SOX2 Protein between Lung Adenocarcinoma and SCC Development Phases As Revealed by Immunohistochemical Analyses

**Figure 2:**
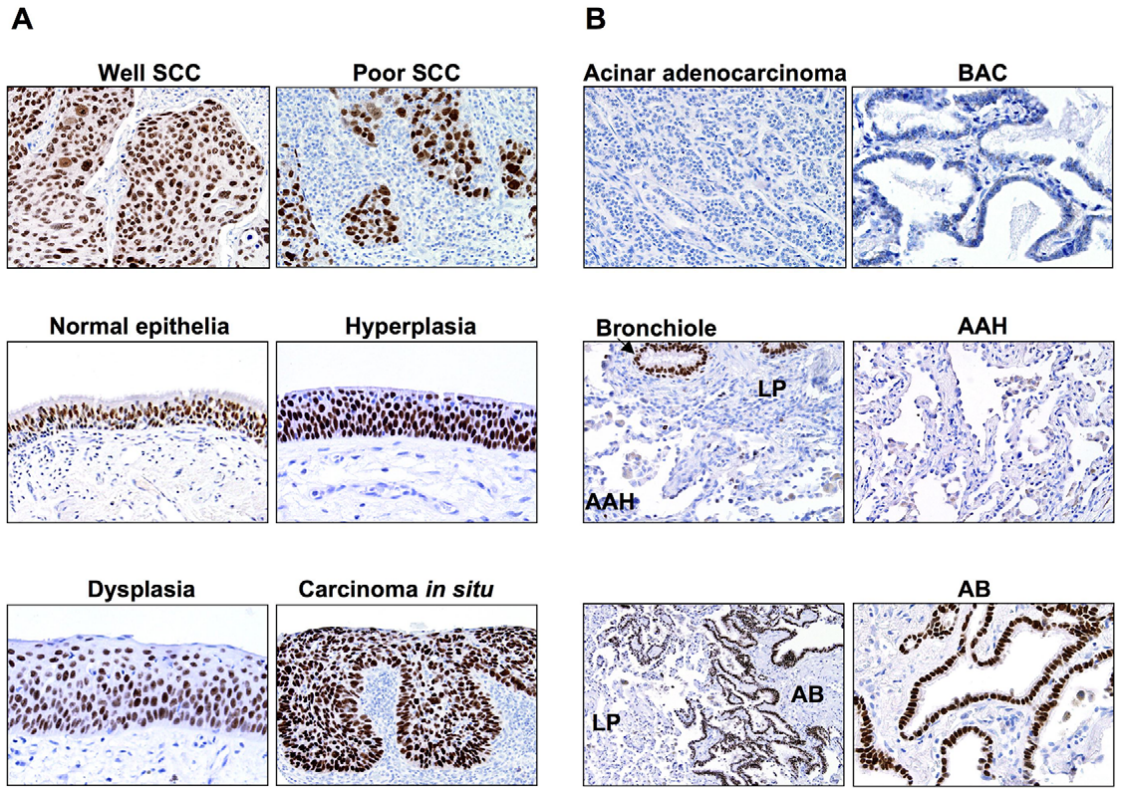
SOX2 protein is highly expressed in the pathogenesis of lung SCC but not of adenocarcinoma. **A.** Representative photomicrographs displaying the immunohistochemical expression of SOX2 protein in histological tissue sections of normal bronchial epithelia, preneoplastic lesions representing SCC development (Hyperplasia, Dysplasia and carcinoma *in situ*), and in well (Well SCC) and poorly (Poor SCC) differentiated SCCs. **B.** Representative photomicrographs of SOX2 expression in lung parenchyma (LP), atypical adenomatous hyperplasia (AAH), alveolar bronchiolization structures (AB), and in acinar adenocarcinoma and bronchioalveolar carcinoma (BAC).

We next attempted to analyze SOX2 expression at the protein level in histological tissue specimens representing different stages in the pathogenesis of lung adenocarcinomas and SCCs. Strikingly, SOX2 protein expression was absent in all normal alveoli (n = 52) and AAH (n = 37) (Figure 2 and Table 1). In sharp contrast, SOX2 positive expression was mainly nuclear and evident in all normal bronchial epithelia (n = 52), all four alveolar bronchiolization structures and in all (n = 32) but one cases of dysplasia and carcinoma *in situ* representing the sequence of SCC pathogenesis (Figure 2 and Table 1).

**Table 1:** SOX2 expression in normal and preneoplastic lung lesions.

Tissue type	N	Immunohistochemistry Expression	
		Positive	Negative
**Normal bronchial epithelia**	52	52	0
**Normal alveoli**	52	0	52
**Atypical adenomatous hyperplasia (AAH)**	37	0	37
**Alveolar bronchiolization**	4	4	0
**Squamous dysplasia and carcinoma ** ***in situ***	32	31	1

### Elevated Expression of SOX2 DNA and Protein in Lung SCCs Relative to Adenocarcinomas

**Figure 3:**
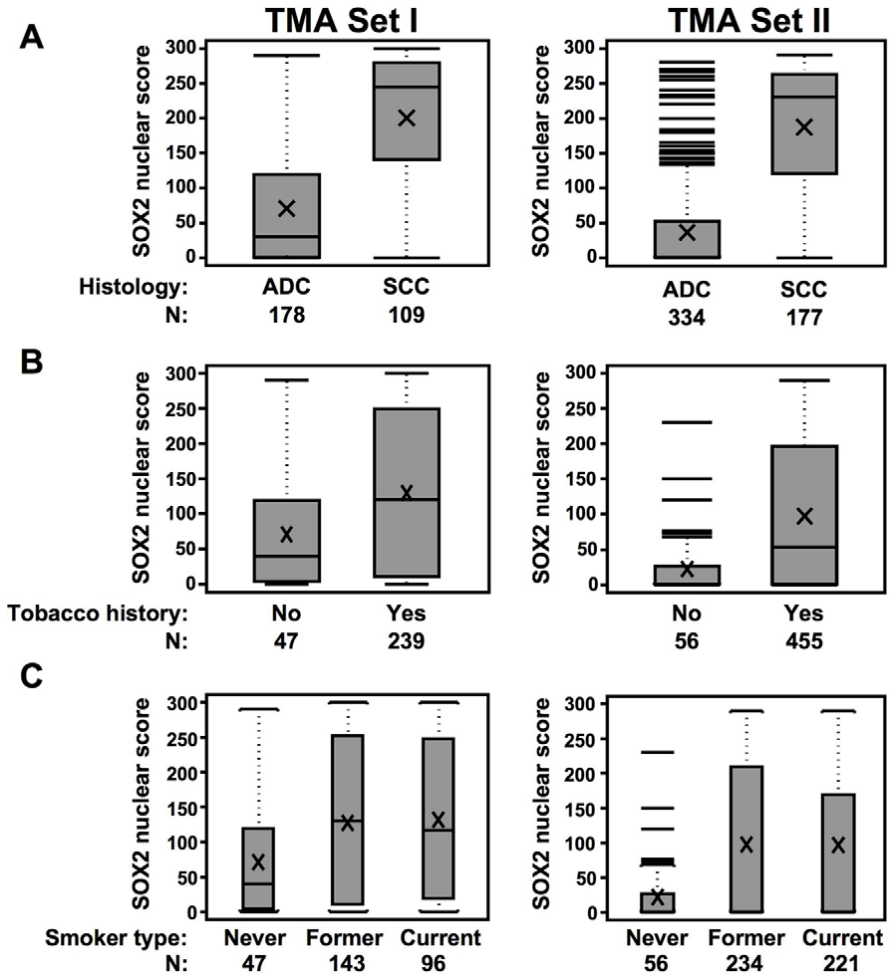
Increased SOX2 expression in lung SCCs compared to adenocarcinomas and its association with smoking patterns. Box-plot depicting statistical analysis by the Wilcoxon-rank test of differences in nuclear SOX2 protein score in both TMA sets I and II between lung adenocarcinomas (ADC) and SCCs (**A**), ever and never NSCLC smokers (**B**) and between never, former and current smokers (**C**).

We next sought to analyze SOX2 protein expression in NSCLC tissue microarrays. The characteristics of the NSCLC patients from which the tissue specimens were obtained are summarized in Table S2. Nuclear SOX2 levels were largely and statistically significantly higher in lung SCCs (n = 109, set I; n = 177 set II) relative to the levels in adenocarcinomas (set I, n = 178; set II, n = 334) (both p<0.001) (Figure 3A). Notably, the median level of SOX2 protein was 8.2 and 39.7 higher in lung SCCs relative to adenocarcinomas when analyzed in TMA sets I and II, respectively (set I, adenocarcinoma, 30, SCC, 245; set II, adenocarcinoma 5.8, SCC, 230). In addition and irrespective of tumor histology, SOX2 protein expression was statistically significantly higher in ever compared to never smokers (set I, p = 0.002; set II, p<0.001) (Figure 3B) and was also significantly increased in current or former smokers compared to never smoker NSCLC patients (set I, p = 0.008; set II, p<0.001) (Figure 3C). Furthermore, in analysis of both TMA sets, SOX2 protein expression was comparable between former and current smoker NSCLC patients.

We also assessed for the levels of *SOX2* DNA copy number in 57 NSCLC tumors. We found 20% SCCs had gene copy gain (8/40; 6/29 with high SOX2 protein expression and 2/11 with low SOX2 expression) (Table S3). In contrast, none of the 17 adenocarcinomas tested displayed *SOX2* gene copy gain with most cases (83%) exhibiting *SOX2* DNA RQs<1.

## Discussion

In this study, we sought to investigate the expression patterns of *SOX2* in NSCLC pathogenesis based on its role and function in the developing and adult mouse lung and trachea [8], [9]. We found that *SOX2* mRNA levels are significantly higher in lung SCCs relative to adenocarcinomas or large-cell lung carcinomas from various published microarray datasets. Moreover, a previously characterized *OCT4/SOX2/NANOG* embryonic stem cell expression signature [11] effectively separated SCCs from lung adenocarcinomas when analyzed in publicly available NSCLC microarray datasets. In addition, we found that SOX2 protein was completely absent in lung adenocarcinoma pathogenesis and highly expressed in SCC development. Lastly, we demonstrated that SOX2 protein expression was largely significantly elevated in lung SCCs relative to adenocarcinomas following analyses of two independent TMA sets and copy gain of the gene was evident in 20% of lung SCCs studied and absent in adenocarcinomas. These findings entail the possible clinical application of *SOX2* expression as a diagnostic biomarker to discriminate lung SCCs from adenocarcinomas, in an analogous fashion to the potential application of *TITF1* as a biomarker for lung adenocarcinomas but not SCCs [2], [6].

It is noteworthy that the *OCT4/SOX2/NANOG* signature is comprised of genes under direct transcriptional control by the *SOX2* transactivating factor [11]. Given the effective separation of lung SCCs and adenocarcinomas by this signature, it is possible that other genes directly or indirectly related to *SOX2* signaling are also dissimilar in expression between both NSCLC subtypes. In accordance and based on analysis of the expression signature in two independent microarray datasets, we validated and demonstrated the correlative expression of both SOX2 and FGFR2 protein in lung SCC tissue histological specimens. Interestingly, *FGFR2* has been shown to be critical for the formation of the lung bud, which typically is associated with *SOX2* expression and *TITF-1* repression, and mice lacking this gene do not form lungs [9], [14], [15]. It is plausible to suggest that the reduction in SOX2 expression in adenocarcinomas of the lung may be evolutionarily conserved.

While completing our study, Bass *et al* reported an amplification of *SOX2* in esophageal (15%) and lung SCCs (23%) [16] assessed by Affymetrix single nucleotide polymorphism (SNP) arrays. Similarly, we found copy gain of the *SOX2* gene in 20% of lung SCCs as revealed by our quantitative PCR analysis. Notably, we also analyzed a set of lung adenocarcinomas and found no significant *SOX2* copy number gain further demonstrating a cell-lineage expression pattern for *SOX2* specifically in lung SCCs. In contrast to the study by Bass *et al*, we also assessed SOX2 protein levels in lung SCCs and adenocarcinomas using two independent and large tissue microarrays and correlated the expression of SOX2 protein expression and copy number gain in 57 NSCLC samples. It is note worthy that several cases displayed discordant SOX2 protein and copy number gain. It is reasonable to suggest that SOX2 expression may be regulated at different levels; at the DNA, mRNA and protein and that it may be important to assess for its expression at all three levels.

In conclusion, we demonstrated a vast increase in expression of the pluripotent stem cell related transcriptional factor, *SOX2*, in squamous cell carcinomas relative to adenocarcinomas of the lung. Moreover, our findings raise the possibility of the activation of SOX2-dependent stem cell-related pathways in squamous cell carcinomas of the lung.

## Materials and Methods

### Analysis of *SOX2* mRNA in Published Microarray Datasets and Integration of the *OCT4/SOX2/NANOG* Signature

The integrated cancer microarray database and data-mining platform, Oncomine [17], was utilized to analyze the expression of *SOX2* in publicly available microarray datasets of human lung carcinomas available on-line. *SOX2* mRNA (probe sets 213721_at and 228038_at) was also compared by microarray analysis between formalin-fixed paraffin embedded (FFPE) lung SCCs and adenocarcinomas (unpublished observations). Genes characterizing an *OCT4/SOX2/NANOG* expression signature [11] were compiled and queried using NetAffx™ from Affymetrix (http://www.affymetrix.com/analysis/index.affx) to search for corresponding probe set annotations in the HG-U95A and HG-U133 plus 2.0 platforms used in the lung cancer microarray studies by Bhattacharjee *et al.* [12] and Bild *et al.* [13], respectively. The *OCT4/SOX2/NANOG* signature gene features were then analyzed alone in published human gene expression data obtained from the reports by Bhattacharjee *et al*. and Bild *et al*. Raw microarray data files from the two published datasets were imported and analyzed using the BRB-ArrayTools v.3.7.0 developed by Dr. Richard Simon and BRB-ArrayTools Development Team [18]. Gene expression data were normalized by Robust multi-array analysis (RMA) in R language environment [19] and median-centered across all samples in each data set before hierarchical clustering analysis. Clustering by average linkage was performed with Cluster 2.11, and results were visualized with TreeView programs (Michael Eisen Laboratory, Lawrence Berkeley National Laboratory and University of California, Berkeley; http://rana.lbl.gov/EisenSoftware.htm). Principal component analysis following gene centering was performed using the BRB-ArrayTools software.

### Immunohistochemistry Analysis, Human Lung Tissues and Tissue Microarray Sets I and II

Detailed description of the normal and preneoplastic histological tissue specimens analyzed and two TMA sets is available in Methods S1. Cytoplasmic FGFR2 protein expression was available based on previous analysis [20]. Details of the immunohistochemical analysis for SOX2 protein expression are also available in Methods S1.

### DNA Extraction and Quantitative PCR (qPCR)

Tumor tissues (40 SCCs, 17 adenocarcinomas) were dissected from FFPE Hematoxylin-stained tissue sections using manual microdissection to ensure that tumor cell proportions are greater than 70% for subsequent DNA extraction. Tumor DNA was extracted using the PicoPure DNA extraction Kit (Arcturus, Mt View, CA) according to the manufacturer's instructions. Five µl of DNA was added to a 20 µl final volume reaction mixture consisting of 10 µl Power SYBR® Green PCR Master Mix (Applied Biosystems, Foster City, CA) and 0.5 µmol/l of each of forward and reverse primers which span 102 dinucleotides (613 to 714) of the *SOX2* gene (ID: NM 003106) as follows: 5′- GAACCCCAAGATGCACAACTC and 5′-CGCTTAGCCTCGTCGATGAAC. *β-*actin was used as an endogenous reference gene (TaqMan® Control Human Genomic DNA, Applied Biosystems) and was amplified as a standard control for calibration. All samples and standard DNA reactions were carried out in triplicates. qPCR was performed using an ABI 7300 Real Time PCR System Sequence (Applied Biosystems) at 50°C for 2 min, 95°C for 10 min, followed by 40 cycles at 95°C for 15 s and 60°C for 1 min. The quantity of the target genes were normalized using the level of the *β-actin* gene, and expressed as relative quantities (RQ) compared with the value of the Human Genomic DNA. RQ equal or larger than 2 was considered as gene copy gain.

### Statistical Analyses

The data were summarized using standard descriptive statistics. The rank-based non-parametric Wilcoxon rank-sum test and the Kruskal-Wallis test were used to assess the statistical significance of the differences in nuclear SOX2 staining intensity score between lung SCCs and adenocarcinomas and based on tobacco history (ever vs never smokers) and type of smokers (never vs former vs current). All tests were two sided. p-values <0.05 were considered statistically significant.

## Supporting Information

Methods S1 Supplementary methods.
(0.04 MB DOC)

Figure S1 Increased expression of SOX2 mRNA in lung SCCs relative to adenocarcinomas in FFPE NSCLC specimens. SOX2 levels were analyzed from microarray analysis of FFPE NSCLC specimens using the Affymetrix HG-U133A platform. P-values were obtained by the Student's t-test.
(0.22 MB TIF)

Figure S2 Correlation of expression of SOX2 and FGFR2 protein in lung SCC tissue specimens. SOX2 and FGFR2 protein levels were assessed by immunohistochemistry as described in Methods S1. Assessment of significance in correlation between SOX2 nuclear and FGFR2 cytoplasmic protein levels was performed using the Spearman Rank correlation test.
(0.31 MB TIF)

Table S1 Separation of 111 NSCLCs from the study of Bild et al. by the OCT4/SOX2/NANOG signature. *P-value was obtained by Fisher's exact test. Sensitivity (probability for an SCC sample to be correctly predicted as SCC) = 0.811. Specificity (probability for an ADC sample to be correctly predicted as an ADC) = 0.776.
(0.03 MB DOC)

Table S2 Patient characteristics in tissue microarray sets I and II.
(0.05 MB DOC)

Table S3 Analysis of SOX2 gene copy gain in lung SCCs and adenocarcinomas and its correlation with its corresponding protein levels. CN, SOX2 gene copy number assessed as relative quantities (RQs) to β-actin.
(0.03 MB DOC)
