# Supplementary material for: Sex Determining Region Y-Box 2 (SOX2) Is a Potential Cell-Lineage Gene Highly Expressed in the Pathogenesis of Squamous Cell Carcinomas of the Lung
Source: PLoS One. 2010 Feb 9;5(2):e9112. doi: 10.1371/journal.pone.0009112 (PMC2817751; doi:10.1371/journal.pone.0009112)
Supplement: Methods S1 — Supplementary methods. (0.04 MB DOC) [file pone.0009112.s001.doc]

**Sex determining region Y-box 2 (SOX2) is a potential cell-lineage gene highly expressed in the pathogenesis of squamous cell carcinomas of the lung**

Ping Yuan^1*^, Humam Kadara^2*^, Carmen Behrens^2^, Xi Ming Tang^2^, Denise Woods^1^, Luisa M. Solis^1^, Jiaoti Huang^4^, Monica Spinola^5^, Wenli Dong^3^, Guosheng Yin^3^, Junya Fujimoto^2^, Edward Kim^2^, Yang Xie^6^, Luc Girard^5^, Cesar Moran^1^, Waun Ki Hong^2^, John D. Minna^5,7,8^ and Ignacio I. Wistuba^1,2†^.

**Supplementary Methods**

**Human lung tissues and tissue microarray (TMA) sets I and II**

For this study, we obtained archived formalin-fixed and paraffin-embedded (FFPE) samples from surgically resected lung cancer specimens containing tumor and adjacent lung tissues from the lung cancer Specialized Program of Research Excellence (SPORE) tissue bank at The University of Texas M. D. Anderson Cancer Center (Houston, TX). The tissue specimens originally had been collected between 1997 and 2005 and had been classified using the 2004 World Health Organization (WHO) classification system [1]. We selected specimens to construct two TMAs sets. The TMA set I comprises 287 NSCLC specimens (178 lung adenocarcinomas and 109 SCCs) obtained from patients who underwent surgery at M.D. Anderson Cancer Center from 1997-2003, and the TMA set II is comprised of 511 NSCLC tumor specimens (334 lung adenocarcinomas and 177 SCCs) obtained from patients who underwent surgery at the same institution from 2003-2005. Details of both sets are available in supplementary table 2. This study was approved by the M. D. Anderson Cancer Center institutional review board. Detailed clinical and pathologic information was available for most of these cases and included patients’ demographic data, smoking history (never smokers or ever smokers, patients who had smoked at least 100 cigarettes in their lifetime), pathologic tumor-node-metastasis (TNM) staging [2], overall survival (OS) time, and recurrence-free survival (RFS) time. Specimens resected from NSCLC stages I-IV patients who had no prior chemotherapy or radiotherapy were used for TMA analysis by immunohistochemistry. After histological examination of NSCLC specimens, the NSCLC TMAs were constructed by obtaining three 1-mm in diameter cores from each tumor at three different sites (periphery, intermediate and central tumor sites). The TMAs were prepared with a manual tissue arrayer (Advanced Tissue Arrayer ATA100, Chemicon International, Temecula, CA).

To assess the immunohistochemical expression of SOX2 in the early pathogenesis of NSCLC, we studied FFPE material placed in TMAs from 52 normal bronchial epithelia, 61 bronchial hyperplasias, 32 squamous dysplasia and carcinomas *in situ* (CIS), as well as 52 normal alveoli, 37 atypical adenomatous hyperplasia and four cases of alveolar bronchiolization. All epithelial lesions were classified according to the World Health Organization (WHO) 2004 criteria. For the epithelial foci TMAs, we used single 2-mm cores to capture most of the preneoplastic lesions.

**Immunohistochemistry analysis.** Immunohistochemistry was performed on histological sections of formalin-fixed paraffin-embedded tissue samples, using the purified primary goat polyclonal anti-human SOX2 antibody (R&D System, Minneapolis, MN) at a dilution of 1:50. The sections were deparaffinized, hydrated, subjected to antigen retrieval by Decloaking Chamber (Biocare Medical, Concord, CA) for 120° C 30 second, 90° C, 10 second with 10 mM sodium citrate (pH 6.0). Peroxide blocking was done with 3% H_2_O_2_ in methanol at room temperature for 15 min, following by 10% fetal bovine in TBS-Tween for 35 min. Sections were then washed with Tris-containing buffer and incubated for 90 min with the primary anti-SOX2 antibody at room temperature. Subsequently, the sections were washed and incubated with Biotinylated Link Universal and Streptavidin AP for 30 min (LSAB+, DAKO) and development with diaminobenzidine chromogen for 5 min. Finally, the sections were rinsed in distilled water, counterstained with hematoxylin (DAKO), and mounted on glass slides prior to evaluation under the microscope. Two lung cancer pathologists examined the SOX2 immunostaining using light microscopy (P.Y. and I.I.W.). Only nuclear SOX2 expression was evaluated. Nuclear SOX2 immunostaining was quantified using a four-value intensity score (0, 1+, 2+, and 3+) and the percentage (0-100%) of the extent of reactivity. The final score was then obtained by multiplying the intensity and reactivity extension values (range, 0-300).

**References**

1. Travis WD, Brambilla E, Muller-Hermelink HK, Harris CC (2004) Tumors of the lung. In: Travis WD, Brambilla E, Muller-Hermelink HK, Harris CC, editors. Pathology and genetics: tumors of the lung, pleura, thymus and heart. Lyon: IARC. pp. 9–124.

2. Mountain CF, Dresler CM (1997) Regional lymph node classification for lung cancer staging. Chest 111: 1718-1723.
