# Supplementary figures and images for: Sex Determining Region Y-Box 2 (SOX2) Is a Potential Cell-Lineage Gene Highly Expressed in the Pathogenesis of Squamous Cell Carcinomas of the Lung
Source: PLoS One. 2010 Feb 9;5(2):e9112. doi: 10.1371/journal.pone.0009112 (PMC2817751; doi:10.1371/journal.pone.0009112)

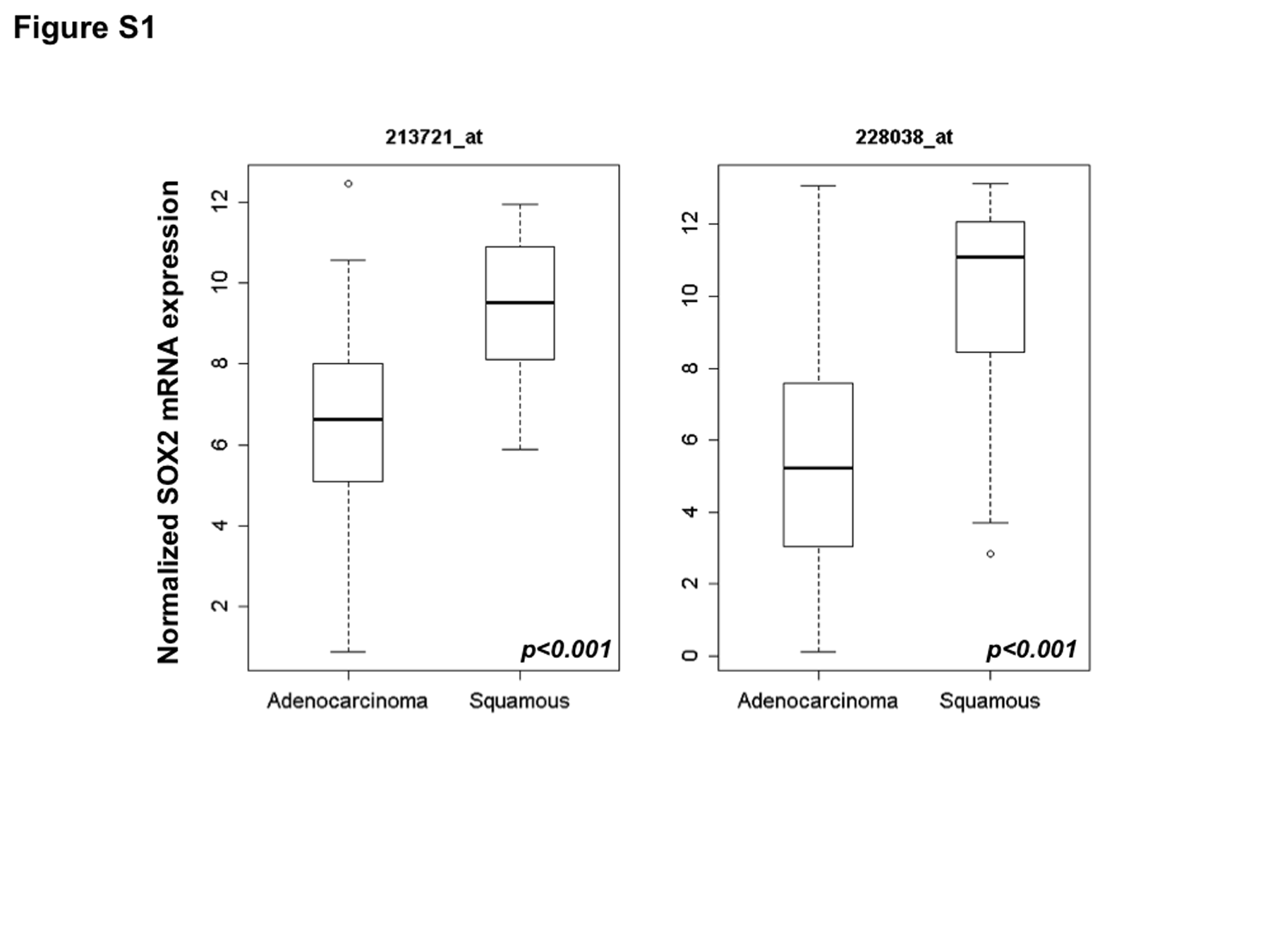

Supplement: Figure S1 — Increased expression of SOX2 mRNA in lung SCCs relative to adenocarcinomas in FFPE NSCLC specimens. SOX2 levels were analyzed from microarray analysis of FFPE NSCLC specimens using the Affymetrix HG-U133A platform. P-values were obtained by the Student's t-test. (0.22 MB TIF) [file pone.0009112.s002.tif]

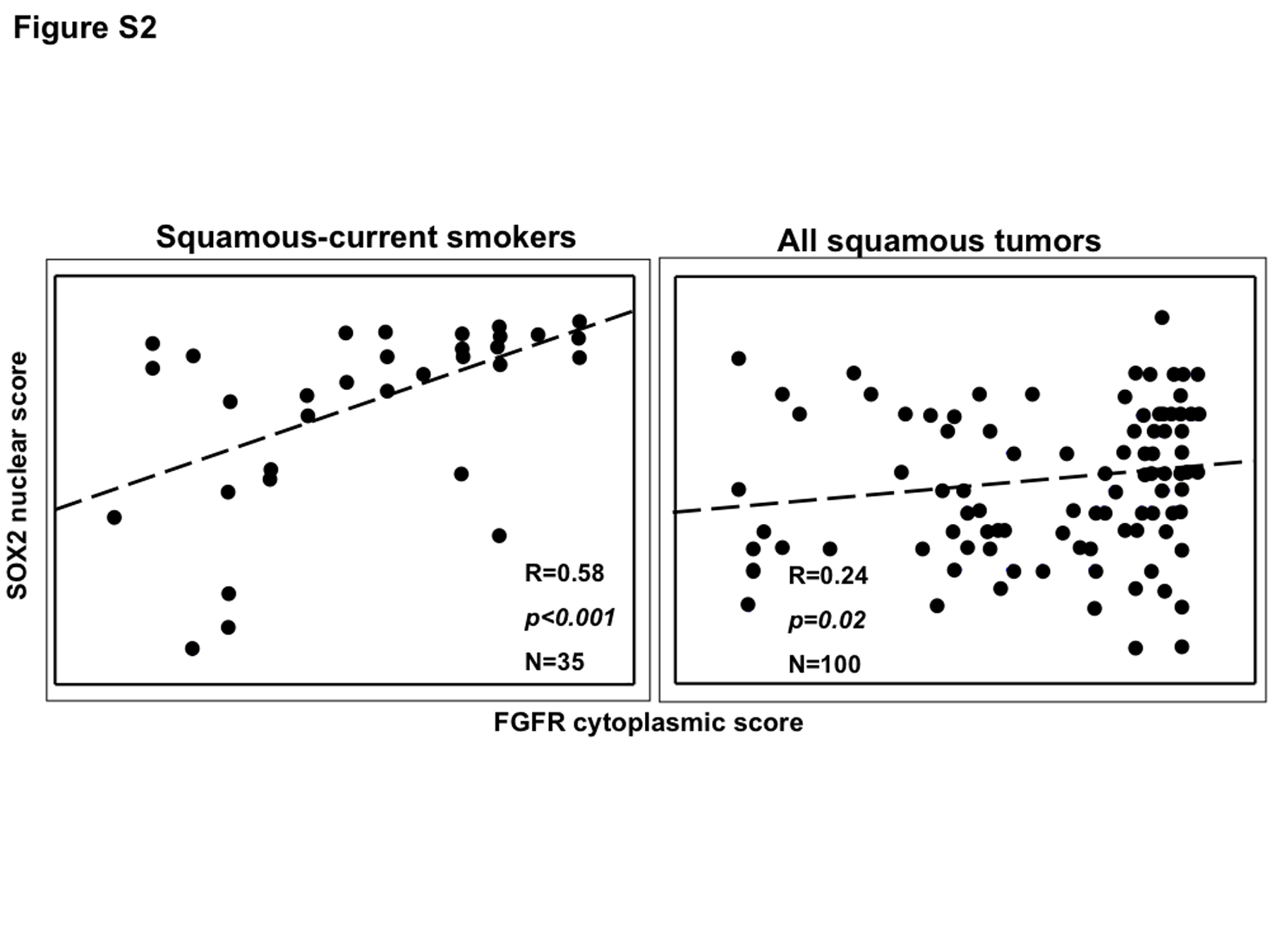

Supplement: Figure S2 — Correlation of expression of SOX2 and FGFR2 protein in lung SCC tissue specimens. SOX2 and FGFR2 protein levels were assessed by immunohistochemistry as described in Methods S1. Assessment of significance in correlation between SOX2 nuclear and FGFR2 cytoplasmic protein levels was performed using the Spearman Rank correlation test. (0.31 MB TIF) [file pone.0009112.s003.tif]
