# Supplementary material for: Sex Determining Region Y-Box 2 (SOX2) Is a Potential Cell-Lineage Gene Highly Expressed in the Pathogenesis of Squamous Cell Carcinomas of the Lung
Source: PLoS One. 2010 Feb 9;5(2):e9112. doi: 10.1371/journal.pone.0009112 (PMC2817751; doi:10.1371/journal.pone.0009112)
Supplement: Table S1 — Separation of 111 NSCLCs from the study of Bild et al. by the OCT4/SOX2/NANOG signature. *P-value was obtained by Fisher's exact test. Sensitivity (probability for an SCC sample to be correctly predicted as SCC) = 0.811. Specificity (probability for an ADC sample to be correctly predicted as an ADC) = 0.776. (0.03 MB DOC) [file pone.0009112.s004.doc]

**Table S1.** Separation of 111 NSCLCs from the study of Bild *et al* by the *OCT4/SOX2/NANOG* signature.

| Cluster | SCC | ADC | P value* |
| --- | --- | --- | --- |
| 1 (*SOX2* high) | 43 | 13 | < 0.001 |
| 2 (*SOX2* low) | 10 | 45 |
