# Supplementary material for: Sex Determining Region Y-Box 2 (SOX2) Is a Potential Cell-Lineage Gene Highly Expressed in the Pathogenesis of Squamous Cell Carcinomas of the Lung
Source: PLoS One. 2010 Feb 9;5(2):e9112. doi: 10.1371/journal.pone.0009112 (PMC2817751; doi:10.1371/journal.pone.0009112)
Supplement: Table S2 — Patient characteristics in tissue microarray sets I and II. (0.05 MB DOC) [file pone.0009112.s005.doc]

**Table S2.** Patient characteristics in tissue microarray sets I and II.

| **Variable** | **Subtype** | **TMA Set I** | | **TMA Set II** | |
| --- | --- | --- | --- | --- | --- |
|  |  | **N** | % | N | % |
| Histology | Adenocarcinoma | 178 | 62.0 | 334 | 65.4 |
|  | Squamous cell carcinoma | 109 | 38.0 | 177 | 34.6 |
| Gender | Female | 152 | 53.0 | 256 | 50.1 |
|  | Male | 135 | 47.0 | 255 | 49.9 |
| Tobacco history | No | 47 | 16.4 | 56 | 11.0 |
|  | Yes | 239 | 83.6 | 455 | 89.0 |
| Smoker type | Current | 96 | 33.6 | 221 | 43.2 |
|  | Former | 143 | 50.0 | 234 | 45.8 |
|  | Never | 47 | 16.4 | 56 | 11.0 |
| Pathological T stage | T1 | 101 | 35.2 | 183 | 35.8 |
|  | T2 | 145 | 50.5 | 259 | 50.7 |
|  | T3/T4 | 41 | 14.3 | 69 | 13.5 |
| Pathological N stage | N0 | 198 | 69.0 | 350 | 68.8 |
|  | N1/N2/N3/Nx | 89 | 31.0 | 159 | 31.2 |
| Pathological M stage | M0 | 278 | 96.9 | 482 | 96.0 |
|  | M1 | 9 | 3.1 | 20 | 4.0 |
| Pathological stage | I | 172 | 59.9 | 289 | 56.6 |
|  | II | 58 | 20.2 | 91 | 17.8 |
|  | III/IV | 57 | 19.9 | 131 | 25.6 |
| Perioperative therapy | No | 231 | 80.5 | 241 | 47.2 |
|  | Yes | 56 | 19.5 | 270 | 52.8 |
