# Supplementary material for: Sex Determining Region Y-Box 2 (SOX2) Is a Potential Cell-Lineage Gene Highly Expressed in the Pathogenesis of Squamous Cell Carcinomas of the Lung
Source: PLoS One. 2010 Feb 9;5(2):e9112. doi: 10.1371/journal.pone.0009112 (PMC2817751; doi:10.1371/journal.pone.0009112)
Supplement: Table S3 — Analysis of SOX2 gene copy gain in lung SCCs and adenocarcinomas and its correlation with its corresponding protein levels. CN, SOX2 gene copy number assessed as relative quantities (RQs) to β-actin. (0.03 MB DOC) [file pone.0009112.s006.doc]

**Table S3.** Analysis of *SOX2* gene copy gain in lung SCCs and adenocarcinomas and its correlation with its corresponding protein levels.

|  |  | ***SOX2* Copy Number** | | |
| --- | --- | --- | --- | --- |
| **SOX2 Protein High** | **N** | **CN<2** | **4>CN>2** | **CN>4** |
| **SCC (score>270)** | 29 | 8 | 15 | 6 |
| **AC (score>193)** | 9 | 6 | 3 | 0 |
| **SOX2 Protein Low** |  |  |  |  |
| **SCC (score<140)** | 11 | 6 | 3 | 2 |
| **AC (score<10)** | 8 | 8 | 0 | 0 |
